# Systemically administered anti-TNF therapy ameliorates functional outcomes after focal cerebral ischemia

**DOI:** 10.1186/s12974-014-0203-6

**Published:** 2014-12-12

**Authors:** Bettina Hjelm Clausen, Matilda Degn, Nellie Anne Martin, Yvonne Couch, Leena Karimi, Maria Ormhøj, Maria-Louise Bergholdt Mortensen, Hanne Birgit Gredal, Chris Gardiner, Ian I L Sargent, David E Szymkowski, Géraldine H Petit, Tomas Deierborg, Bente Finsen, Daniel Clive Anthony, Kate Lykke Lambertsen

**Affiliations:** Department of Neurobiology Research, Institute of Molecular Medicine, University of Southern Denmark, J.B. Winsloewsvej 21, st., 5000 Odense, Denmark; Department of Diagnostics, Molecular Sleep Laboratory, Glostrup Hospital, Nordre Ringvej 69, 2600 Glostrup, Denmark; Department of Pharmacology, University of Oxford, Mansfield Road, OX1 3QT Oxford, UK; Nuffield Department of Obstetrics and Gynecology, University of Oxford, Headley Way, OX1 3QT Oxford, UK; Department of Clinical Sciences, Laboratory for Experimental Medical Science, Neuronal Survival Unit, 22100 Lund University, BMC B11, Sölveg 19, Lund, Sweden; Xencor Inc, 111 W Lemon Ave, Monrovia, CA 91016 USA; Department of Veterinary Clinical and Animal Sciences, Facuty of Health and Medical Sciences, University of Copenhagen, Dyrlægevej 16, 1870 Frederiksberg, Denmark

**Keywords:** SolTNF and tmTNF, Granulocytes, Behavior, Acute phase response, Microvesicle, Inflammation

## Abstract

**Background:**

The innate immune system contributes to the outcome after stroke, where neuroinflammation and post-stroke systemic immune depression are central features. Tumor necrosis factor (TNF), which exists in both a transmembrane (tm) and soluble (sol) form, is known to sustain complex inflammatory responses associated with stroke. We tested the effect of systemically blocking only solTNF versus blocking both tmTNF and solTNF on infarct volume, functional outcome and inflammation in focal cerebral ischemia.

**Methods:**

We used XPro1595 (a dominant-negative inhibitor of solTNF) and etanercept (which blocks both solTNF and tmTNF) to test the effect of systemic administration on infarct volume, functional recovery and inflammation after focal cerebral ischemia in mice. Functional recovery was evaluated after one, three and five days, and infarct volumes at six hours, 24 hours and five days after ischemia. Brain inflammation, liver acute phase response (APR), spleen and blood leukocyte profiles, along with plasma microvesicle analysis, were evaluated.

**Results:**

We found that both XPro1595 and etanercept significantly improved functional outcomes, altered microglial responses, and modified APR, spleen T cell and microvesicle numbers, but without affecting infarct volumes.

**Conclusions:**

Our data suggest that XPro1595 and etanercept improve functional outcome after focal cerebral ischemia by altering the peripheral immune response, changing blood and spleen cell populations and decreasing granulocyte infiltration into the brain. Blocking solTNF, using XPro1595, was just as efficient as blocking both solTNF and tmTNF using etanercept. Our findings may have implications for future treatments with anti-TNF drugs in TNF-dependent diseases.

**Electronic supplementary material:**

The online version of this article (doi:10.1186/s12974-014-0203-6) contains supplementary material, which is available to authorized users.

## Background

Tumor necrosis factor (TNF) is an immunomodulatory molecule known to be implicated in central nervous system (CNS) insults such as stroke [1]. Immune responses within the CNS, as well as systemic inflammatory events, play important roles in the progression, repair and recovery of stroke, offering new immune-based approaches as future treatment strategies in stroke patients. TNF is present in low concentrations in normal brain tissue and upregulated after ischemia [1]. It exists both as transmembrane (tm)TNF and soluble (sol)TNF. tmTNF acts through cell-to-cell contact to promote juxtacrine signaling and is important for cellular communication in the innate immune system [2], but also for functional recovery and axonal preservation [3], whereas solTNF acts in a paracrine manner and is an important mediator of both acute and chronic inflammation [4].

Anti-TNF therapies such as etanercept, which blocks both solTNF and tmTNF, are currently used to treat chronic inflammatory diseases [5,6], and appear to relieve fatigue and symptoms of depression associated with chronic diseases [5]. Furthermore, peri-spinal etanercept has been used with success in stroke and traumatic brain injury patients, where treatment resulted in neurological improvement [7,8]. However, their use is hampered by side effects, including increased risk of sepsis, demyelinating disease, neuropathies, heart failure and also infections [9], which represents a considerable risk for stroke patients. Since etanercept inhibits both solTNF and tmTNF, this raises the possibility that solTNF-specific inhibitors, sparing tmTNF, have the potential to inhibit deleterious inflammation without compromising the immune system’s response to infections. XPro1595, an engineered dominant-negative TNF that inactivates only solTNF [10], has proven to be effective in animal models of CNS disorders involving increased TNF production [3,11,12], and in attenuating experimental arthritis [13] and endotoxin-induced liver injury [14], without suppressing the innate immunity to infection, in contrast to etanercept treatment. The ability of XPro1595 to be tmTNF-sparing and solTNF-selective potentially makes XPro1595 a safer clinical drug than etanercept as it ensures that the role of tmTNF in immune function and myelin preservation is not compromised.

In the present study, we used etanercept and XPro1595 to test the effect of systemic administration on functional recovery, infarct volume, and systemic and central inflammatory responses in a murine model of focal cerebral ischemia.

## Materials and methods

### Animals

Adult male C57BL/6 mice (between seven and eight weeks of age, n = 256) were purchased from Taconic Ltd. (Ry, Denmark) and transferred to the Laboratory of Biomedicine, University of Southern Denmark, where they were allowed to acclimatize for seven days prior to surgery. Animals were housed under diurnal lighting conditions and given free access to food and water. All animal experiments were performed in accordance with the relevant guidelines and regulations approved by the Danish Animal Ethical Committee (numbers 2011/561-1950 and 2013-15-2934-00924).

### Induction of permanent middle cerebral artery occlusion

The distal part of the left middle cerebral artery (MCA) was permanently occluded [15] under Hypnorm and Dormicum anesthesia (fentanyl citrate (0.315 mg/ml; Jansen-Cilag) and fluanisone (10 mg/ml; Jansen-Cilag, Birkerød, Denmark), and midazolam (5 mg/ml; Hoffmann-La Roche, Hvidovre, Denmark)), respectively. After surgery, mice were injected subcutaneously with 1 ml of 0.9% saline and allowed to recover in a 25°C controlled environment. Mice surviving for five days were returned to the conventional animal facility after 24 hours. For post-surgical analgesia, mice were treated with 0.001 mg/20 g buprenorphine hydrochloride (Temgesic, Schering-Plough, Ballerup, Denmark) three times at eight-hour intervals, starting immediately prior to surgery.

### Group size and study design

The size of the ischemic infarct was measured in three separate randomized, double-blinded, vehicle-controlled studies in mice allowed to survive for six hours (n = 30), 24 hours (n = 60) and five days (n = 74) after induction of permanent middle cerebral artery occlusion (pMCAO). In order to evaluate the effect of ischemia on functional outcome and the acute phase response (APR), a group of sham-treated mice were included at all time points (total n = 35). Furthermore, un-manipulated controls were included in flow cytometric and microparticle analyses (total n = 27). A total of 12 mice were excluded due to lack of infarct in mice subjected to pMCAO or presence of unintended infarcts in shams. Mortality was 1.8% and there were no differences in mortality between the different treatment groups.

### Pharmacological treatment

XPro1595 [13] or etanercept (Enbrel, Amgen-Wyeth, Thousand Oaks, CA, USA) were administered intravenously once, at a dose of 10 mg/kg, 30 minutes after surgery. Saline was used as the vehicle. Mice subjected to sham surgery were given an intravenous injection of saline 30 minutes after surgery. The peak concentration in serum (C_max_) after murine intravenous dosing of XPro1595 at 10 mg/kg was 945.7 μg/ml and the terminal half-life was 19.1 hours (data not shown).

### Physiological parameters

Mice were weighed at the time of pre-training, before surgery, and one, three and five days after surgery. Rectal temperature was measured prior to, and 30 minutes and three hours after surgery.

### Behavioral tests

Functional outcomes were evaluated one, three and five days after pMCAO using different behavioral tests designed to detect motor deficits. Prior to behavioral testing, mice were allowed to acclimatize in the behavior room.

#### Grip strength test

The grip strength meter (BIO-GT-3, BIOSEB, Vitrolles, France) was used to study neuromuscular function in mice subjected to pMCAO and sham surgery. The peak amount of force was recorded in five sequential trials and the highest grip value was recorded as the score [16]. We analyzed the grip strength in individual (left and right) front paws prior to (baseline) and one, three and five days after pMCAO. The unit of force measured is presented as grams (g). Asymmetry between paws in individual mice following pMCAO were calculated and are presented as delta (Δ) grip strength measured in grams (g). Mice that were allowed to survive for 24 hours were tested on day one, and mice that were allowed to survive for five days were tested on day three and five.

#### Horizontal rod test

In order to test motor coordination, dynamic balance and asymmetry, mice were placed on the centre of a horizontal rod, located 80 cm above the floor. Mice were allowed to explore and walk the rod for three minutes. The frequency of right and left hind limb slips was recorded and the total distance travelled was tracked using the SMART video tracking software (Panlab, Barcelona, Spain)[17].

#### Rotarod performance test

In order to evaluate drug-induced differences, motor coordination and performance and balance [18], we performed the rotarod test (LE8200, Panlab Harvard Apparatus, Barcelona, Spain). The test comprised a pre-training part prior to surgery (30 seconds at four rotations per minute (rpm)) and a trial part consisting of four trials (T1 to T4) 24 hours or five days after surgery. Mice were placed on the rotarod which was set in accelerating mode. The speed of the rotor was accelerated from 4 to 40 rpm over five minutes. Time spent on the rotarod in each trial for each mouse was recorded.

### Tissue processing

#### Fresh frozen tissue

Mice were killed by cervical dislocation, and brains and livers were quickly removed, frozen in CO_2_ and stored at −80°C until further processing. Blood samples were collected in EDTA-coated Eppendorf tubes, spun twice for 10 minutes at 3,000 g and 4°C, and stored at −80°C until further processing. Brains were cut coronally in six parallel series of 30 μm and liver samples were cut into 30 μm cryostat sections and stored at −80°C until further processing.

#### Perfusion fixed tissue

Mice were deeply anesthetized with an overdose (0.15 ml) of pentobarbital (200 mg/ml) containing lidocaine (20 mg/ml) (Glostrup Apotek, Glostrup, Denmark) and perfused through the left ventricle using 4% paraformaldehyde (PFA), as previously described [19]. Brains from mice with 24 hours survival intended for immunohistochemistry for the granulocyte marker Gr1 (see below) were cut coronally in six parallel series as free-floating 60-μm thick sections and stored in a cryoprotective solution at −12°C (n = 6), or were cut coronally into 12 parallel series as 20-μm thick cryostat sections (n = 5) and stored at −20°C, until further processing.

#### Flow cytometric analysis

Mice were anesthetized intraperitoneally with an overdose of pentobarbital containing lidocaine and perfused through the left ventricle using phosphate-buffered saline (PBS), as previously described [19]. Prior to perfusion, 80 μl blood was collected from each mouse using EDTA-coated capillaries and placed in Hanks’ balanced salt solution (HBSS: 0.14 M NaCl, 5.4 mM KCl, 0.4 mM MgSO4•7H2O, 0.4 mM Na2HPO4(anhydrate), 1.3 mM CaCl22H2O, 4.2 mM NaHCO3, 0.4 mM KH2PO4, 0.5 mM MgCl26H2O, and 5 mM glucose) as previously described [19]. Furthermore, spleen and ipsi- and contralateral cortices were quickly removed and processed as previously described [19].

### Infarct volumetric analysis

Every sixth section was stained with toluidine blue solution (TB: 0.08 M Na_2_HPO_4_•2H_2_O, 0.07 M citric acid, and 0.01% TB (Merck Millipore, Hellerup, Denmark)) for direct infarct volume estimation using the Cavalieri principle, as previously described [15,16]. In addition, in order to correct for edema, the volume of the contralateral and the nonischemic ipsilateral cortex and the volume of injury spanning from 1,080 μm anterior to 1,080 μm posterior of the anterior commissure was compared using an indirect method of infarct volume estimation [16].

### Quantitative PCR

Liver and brain mRNAs were extracted using the RNeasy Mini Kit (Qiagen, Manchester, UK)) according to the manufacturer’s instructions. cDNA was prepared as previously described [16,20] and qPCR analysis was performed using the following conditions: five minutes primer extension at 25°C, followed by 25 minutes reverse transcription at 55°C and finally five minutes enzyme inactivation at 95°C, as previously described [16,20]. Samples were run against standard curves generated from serially diluted cDNA from liver samples obtained from mice subjected to pMCAO. Primer sets were designed by PrimerDesign Ltd. (Southampton, UK) and analyzed using SYBR green as previously described [20]. Primer sets were: serum amyloid A2 (*SAA2*) (forward: TTCATTTATTGGGGAGGCTT and reverse: GCCAGCTTCCTTCATGTCAG), serum amyloid P-component (*SAP*) (forward: CAAGGCGGCAGAGTTCAC and reverse: GGAGAGGATTTTTATTTGGC), Chemokine (C-C motif) ligand 2 (*CCL2*) (forward: TGAAGTTGACCCGTAAATCTGAA and reverse: AGGCATCACAGTCCGAGTC), interleukin (*IL*)*-1β* (forward: TGTAATGAAGACGGCACAC and reverse: TCTTCTTTGGGTATTGCTTGG), Chemokine (C-X-C motif) ligand (*CXCL1*) (forward: GCTGGGATTCACCTCAAGAAC and reverse: TGTGGCTATGACTTCGGTTTG), *CXCL10* (forward: CATCCCGAGCCAACCTTCC and reverse: CACTCAGACCCAGCAGGAT), *IL-10* (forward: AGGACTTTAAGGGTTACT and reverse: AATGCTCCTTGATTTCTG), *iNOS* (forward: GGACAGCACAGAATGTTCCAGAA and reverse: CAAAATCTCTCCACTGCCCCAG), and *TNF* (forward: GCCTCCCTCTCATCAGTTCTAT and reverse: TTTGCTACGACGTGGGCTA). Arg1 primers (Mm00475988_m1) were purchased from Life technologies (Nærum, Denmark). Liver results were reported relative to the expression of the housekeeping gene glyceraldehyde phosphate dehydrogenase (*GAPDH*) [20]. All data were normalized to the corresponding sham group, which at all time points represented a mean value of 1. Brain *TNF*, *IL-1β* and *CD11b* mRNA qPCR analyses were performed as previously described [16].

### Immunohistochemistry

Immunohistochemical staining for TNF was performed using the alkaline phosphatase-conjugated rabbit anti-TNF antibody (Sigma-Aldrich, Brøndby, Denmark) as described in Lambertsen *et al*. [21]. Visualization of the Mac-1 antigen (CD11b; AbDSerotec, Copenhagen, Denmark) on fresh frozen sections and the Gr1 antigen (Ly-6G and Ly-6C, BD Biosciences, Albertslund, Denmark) on free-floating vibratome and perfusion fixated sections was performed with the streptavidin and horseradish peroxidase technique [15,22]. Substitution of the primary antibody with serum immunoglobulin (IgG: DakoCytomation, Glostrup, Denmark) or specific isotype controls gave no signal.

### Western blotting

Total protein was extracted in 1% lysis buffer (RIPA, Merck Millipore, Hellerup, Denmark) containing a soluble protease inhibitor cocktail (Roche Diagnostics, Hvidovre, Denmark) according to Lambertsen *et al*. [16]. Protein concentrations were estimated using the Bradford Protein Quantification method.

Western blotting analysis for TNF (Abcam, Cambridge, UK, 1:2,000) was performed using 20 μg protein extract separated on bis/tris 4-12% SDS-PAGE gels (Nupage™, Invitrogen, Tåstrup, Denmark) essentially as previously described [16]. SeeBlue Plus2 pre-stained standard (Invitrogen) was used as a molecular weight marker and 0.5 ng 17 kDa murine recombinant TNF (Sigma Aldrich) was included as a positive control. Densitometry was performed using Image J analysis software (version 1.47, National Institutes of Health (NIH), Bethesda, Maryland, USA) following recommendations of the Image J developers. Analysis was performed on two independent gels with two mice per group.

### Flow cytometry

Flow cytometry was performed essentially as previously described [16,19] using FACSVerse (BD Biosciences) and data analyzed using the FACSuite software. TNF^+^ microglia (CD11b^+^CD45^dim^), TNF^+^ macrophages (CD11b^+^CD45^high^Gr1^−^) and TNF^+^ granulocytes (CD11b^+^CD45^high^Gr1^+^) were identified as previously detailed [16,19]. Control mice and mice allowed to survive for six and 24 hours after pMCAO were treated intravenously with either saline, XPro1595 or etanercept 30 minutes after surgery.

Prior to fixation, cells were stained for live/dead cells for 30 minutes at 4°C using a Fixable Viability Dye eFluoro 506 (eBioscience, Hatfield, UK) diluted in PBS. A total of 1,000,000 events were collected using forward scatter (FSC) and side scatter (SSC) and analysis of the live/dead gate revealed comparable numbers of dead cells in all the samples. In addition, blood and spleen samples were collected and analyzed for CD45, CD11b, Gr1, and CD3 expression.

Positive staining for TNF (Biolegend, Copenhagen, Denmark), CD11b, CD45, Gr1 and CD3 (BD Pharmingen, Albertslund, Denmark) was determined based on fluorescence levels of the respective isotype controls (Biolegend and BD Pharmingen). The mean fluorescence intensity (MFI) was calculated as the geometric mean of each population in the TNF, CD45 and CD11b positive gates, respectively.

### Estimation of polymorphonucleated cells within the infarcted cortex

The number of polymorphonuclear cells/mm^2^ as a measure for granulocyte infiltration six and 24 hours after pMCAO was estimated based on nuclear morphology using TB-stained sections. In practice, calibrated high-power fields (40×) located within the infarct area, and spanning from 1,080 μm anterior to 1,080 μm posterior of the anterior commissure, were photographed and manually counted by a blinded observer on a minimum of 10 frames from each mouse.

### Microvesicle analysis

In total, 100 μl of plasma was diluted 1:10 in Dulbecco’s sterile filtered PBS (Sigma Aldrich) and centrifuged at 30,000 g for one hour to remove interfering lipoprotein particles. The pellet was resuspended in 100 μl PBS containing 0.1% bovine serum albumin (Sigma Aldrich) diluted 1:10 in PBS immediately prior to analysis. Microvesicle size and concentration were determined by Nanoparticle Tracking Analysis (NTA) using a NS500 analyser equipped with a 488 nm laser and NTA software (Nanosight Ltd, Espoo, Finland) as previously described [23]. Analysis settings were standardized using 100 nm colloidal silica microspheres (100, 150, 300 and 400 nm; Polysciences, Eppelheim, Germany) and these data were used to verify size measurements and calibrate concentration measurements. Five 30-second videos were made for each sample. The sample was advanced with a five-second delay between each recording using the script control facility. The videos were analyzed in *batch process* mode using automatic blur and minimum expected particle size with an automatic detection threshold level 10, after visually checking that the five size profiles on the screen were in concordance.

### Data analysis

Quantitative data are presented as means ± standard error of mean (SEM). Weight and temperature analyses were performed using two-way repeated measures (RM) analysis of variance (ANOVA). Infarct volumetric analysis, qPCR, flow cytometry, grip strength and microvesicle analyses were performed using one-way ANOVA. Grip strength asymmetry and horizontal rod analyses were performed using paired t-tests. Pearson correlation analysis was used to analyze correlations between liver chemokines and between microvesicle counts and infarct volumes. All statistical analyses were followed by the appropriate *post-hoc* test and performed using Prism 6 software for Macintosh (GraphPad software, La Jolla, CA, USA) and considered significant at *P* ≤0.05.

## Results

### Systemically injected anti-TNF therapy does not affect infarct size after permanent focal cerebral ischemia

**Figure 1:**
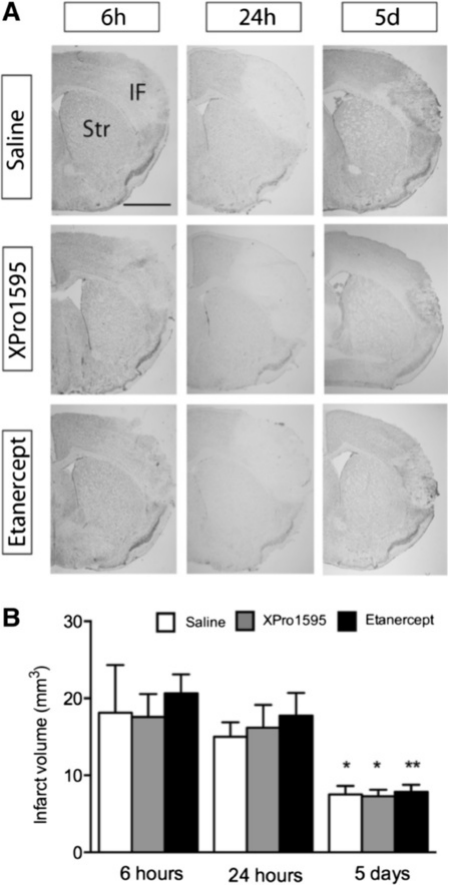
**Systemic anti**-**TNF therapy does not affect infarct volume after focal cerebral ischemia. (A)** Toluidine blue staining of brain sections from mice treated with either saline, XPro1595 or etanercept and allowed to survive for either six hours, 24 hours or five days. Scale bar: 1 mm. **(B)** Direct infarct volume measurements showed no difference in infarct volumes between saline-, XPro1595- and etanercept-treated mice at either six hours ( eight to 15 per group), 24 hours (15 per group) or five days (14 to 17 per group) (one-way ANOVA). A significant drop in infarct size was observed in all three groups five days after pMCAO, compared to six and 24 hours (*P <0.05, **P <0.01). ANOVA, analysis of variance; d, days; h, hours; IF, infarct; pMCAO, permanent middle cerebral artery occlusion; Str, striatum.

Focal cerebral ischemia produced a cortical infarct, which was visible in TB-stained sections at six hours, 24 hours and five days after pMCAO (Figure 1A). Comparison of mean infarct volumes showed that anti-TNF therapy targeting either solTNF using XPro1595, or both solTNF and tmTNF using etanercept, did not affect infarct size at six hours (*P* = 0.79), 24 hours (*P* = 0.76) or five days (*P* = 0.92) after pMCAO (Figure 1B). In line with previous studies [24], we found that in all three groups infarct size had decreased at five days, most likely as a result of edema resolution and resorption of infarcted tissue. Indirect infarct volumetric analysis also revealed no differences in edema formation between the different groups (data not shown).

### XPro1595 and etanercept improve functional outcome after focal cerebral ischemia

In order to distinguish between solTNF- and tmTNF-mediated effects on functional recovery, we evaluated behavior and motor function in mice subjected to pMCAO and treated with either XPro1595 or etanercept. To identify and validate significant behavioral improvements, we also included groups of mice subject to sham surgery.

**Figure 2:**
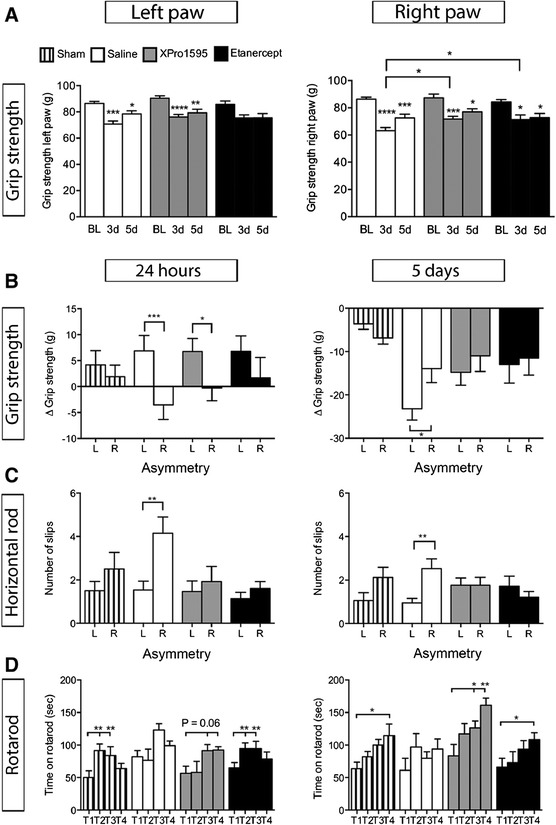
**Anti-** **TNF therapy improves functional outcomes after focal cerebral ischemia. (A)** Neuromuscular function presented as grip strength in grams (g), showing post-surgical weakness in both left and right front paws in saline- and XPro1595-treated mice three and five days after pMCAO compared to baseline grip strength. Etanercept-treated mice showed no loss of grip strength on the left paw, but a significant reduction in grip strength three and five days after pMCAO. BL, baseline. (P < 0.05, ***P < 0.001, ****P < 0.0001 **(B)** Grip strength analysis at 24 hours after pMCAO showed that asymmetry (Δ grip strength) was evident in saline-treated mice (***P <0.001, paired t-test) and, to a lesser extent, in XPro1595-treated mice (*P <0.05), but no asymmetry was observed in etanercept-treated or sham mice (six to 14 per group)(left graph). Grip strength analysis at five days showed that asymmetry was still present in saline-treated mice (*P <0.05) but not in sham, XPro1595- and etanercept-treated mice (13 to 17 per group)(right graph). **(C)** The horizontal rod test showed that only saline-treated mice displayed asymmetry both 24 hours (left graph) and five days (right graph) after pMCAO as saline-treated mice displayed significantly more slips on the right hind limb compared to the left limb after pMCAO (**P <0.01, paired t-test), whereas sham, XPro1595- and etanercept-treated mice did not display any asymmetry (six to 17 per group). **(D)** Assessment of motor function using the rotarod test showed that saline-treated mice subjected to focal cerebral ischemia did not display normal learning skills at 24 hours (left graph) and five days (right graph), whereas both XPro1595- and etanercept-treated mice displayed normal learning skills (T1 to T4) both at 24 hours and five days, comparable to sham mice (*P < 0.05, **P < 0.01, one-way ANOVA, five to six per group. g, grams; L, left; pMCAO, permanent middle cerebral artery occlusion; R, right; T, trial; sec, seconds.

We detected a post-surgical weakness of both the left (L) and right (R) front paws in saline- and XPro1595-treated mice 3 and 5 days after pMCAO compared to normal baseline grip strength (Figure 2A). Etanercept-treated mice showed no difference on the left paw, however a significant reduction on the right paw (Figure 2A). XPro1595- and etanercept-treated mice performed significantly better on day 3 compared to saline-treated mice (Figure 2A). Further, grip strength analysis showed significant pMCAO-induced front paw asymmetry in saline-treated mice 24 h and 5d after pMCAO as compared to sham mice and pre-treatment baseline grip strengths (represented by delta (Δ) values) (Figure 2B). Minor asymmetry was observed in XPro1595-treated mice at 24 h, but not at 5d (Figure 2B). No asymmetry was observed in etanercept-treated mice. The increase in Δ grip strength observed on the left paws in all mice 24 h after pMCAO is likely a result of an increased ”*flight*-*or*-*fight*” response observed at this early time point after surgery [16]. All together, the grip strength data indicate that anti-TNF therapy ameliorates neuromuscular asymmetry normally caused by pMCAO.

The horizontal rod test supported the findings from the grip strength test in saline-treated mice, but showed no asymmetry of the hindlimbs in XPro1595- and etanercept-treated mice 24 h and 5d after pMCAO (Figure 2C). We found no difference in distance travelled (cm) (P = 0.52, data not shown) or speed (cm/sec) (P = 0.82, data not shown) between sham and saline-treated pMCAO mice. Also, distance travelled and speed in anti-TNF-treated mice were comparable to sham and saline-treated mice. The rotarod test showed significantly altered motor learning skills in saline-treated mice 24 h and 5d after pMCAO compared to sham mice, a change which was not observed in XPro1595- and etanercept-treated mice (Figure 2D). These data suggest that XPro1595 and etanercept improved motor learning skills after focal cerebral ischemia.

### Physiological parameters

To exclude the possibility that the improved behavioral effects were merely a result of reduced core temperature, which is neuroprotective [25], we monitored rectal body temperature during and after anti-TNF therapy (Table 1). Results showed that all groups experienced a significant anesthesia-induced drop in rectal temperature 30 min and 3 h after surgery (P < 0.001), an outcome induced by the immobilization of the mice, however there were no differences between treatment groups. Also, no differences were observed in changes in body weights (Δ weight loss (g)) at any time point between treatment groups (Table 1). However, all 4 groups of mice displayed a significant drop in weight from baseline to day 3 (P < 0.0001 for all groups) and day 5 (P < 0.01 for saline, XPro1595 and etanercept); however the weight drop at day 5 was less in the sham group (P < 0.05).

**Table 1:** **Assessment of physiological parameters in saline**-, **XPro1595**- **and etanercept**-**treated mice after focal cerebral ischemia**

**Time point**	**Sham**	**Saline**	**XPro1595**	**Etanercept**
**Weight** **(** **Δg** **)**				
D0	0.69 ± 0.15	0.48 ± 0.16	0.41 ± 0.14	0.45 ± 0.17
D1	0.19 ± 0.17	0.16 ± 0.20	0.12 ± 0.24	0.11 ± 0.21
D3	****-0.61 ± 0.20	****-0.79 ± 0.22	****-0.89 ± 0.15	****-0.81 ± 0.14
D5	*0.14 ± 0.27	**0.11 ± 0.18	**-0.12 ± 0.22	**-0.14 ± 0.29
**Temperature** **(°** **C** **)**				
BL	32.69 ± 0.49	32.21 ± 0.38	31.95 ± 0.32	32.41 ± 0.35
30 min after pMCAO	***27.28 ± 0.22	***27.37 ± 0.22	***27.52 ± 0.15	***27.41 ± 0.21
3 h after pMCAO	***27.15 ± 0.32	***26.89 ± 0.27	***26.51 ± 0.30	***26.59 ± 0.28

*BL*, baseline. Data are presented as mean ± SEM. *P < 0.05, **P < 0.01, ***P < 0.001, ****P < 0.0001 (two-way repeated measures ANOVA).

### Anti-TNF therapy affects microglial activation

We have previously shown that TNF is preferentially produced by microglia (CD11b^+^CD45^dim^ cells) and macrophages (CD11b^+^CD45^high^Gr1^−^ cells) after pMCAO [16,19]. Consequently, we sought to examine whether anti-TNF therapy altered microglial and/or leukocyte responses in the brain.

**Figure 3:**
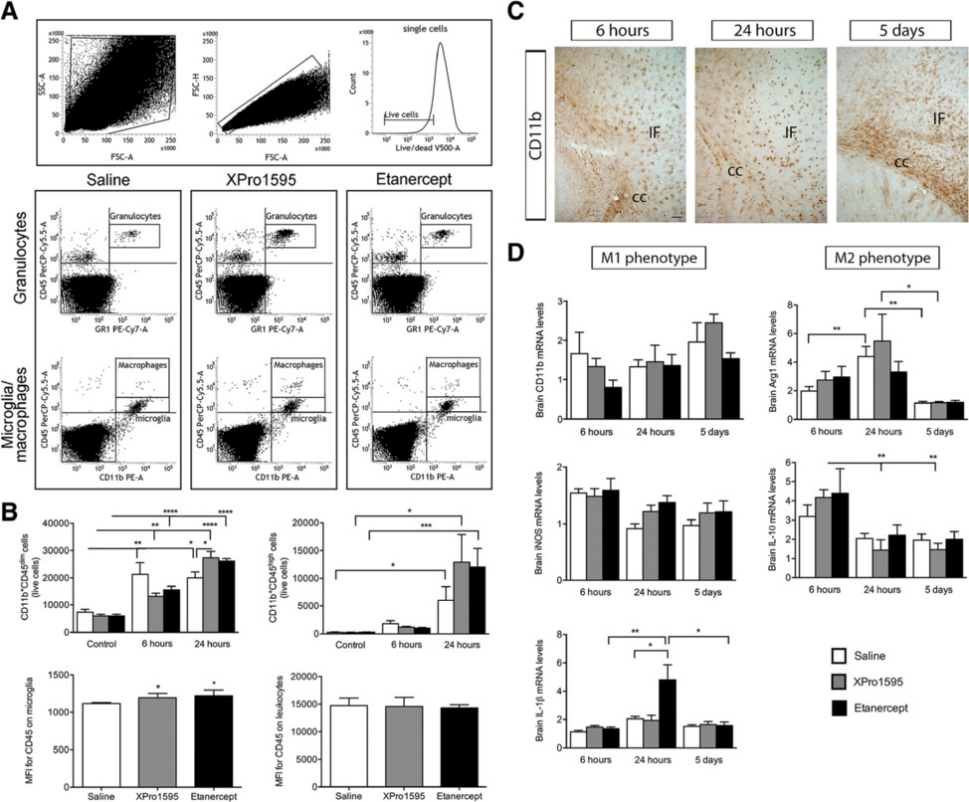
**Analysis of brain microglial/leukocyte responses after anti-** **TNF therapy. (A)** Gating strategy: FSC/SSC was used to define leukocytes, monocytes and granulocytes. Singlet cells were identified using FSC-A/FSC-H, and only live cells were included. Dot plots showing CD45^high^Gr1^+^ granulocytes,, CD45^dim^CD11b^+^ microglia, and CD45^high^CD11b^+^Gr1^-^ macrophages **(B)** Microglial numbers were increased at six and 24 hours in all groups compared to the respective non-lesioned mice. Furthermore, the number of microglia was significantly increased in XPro1595-treated mice compared to saline-treated mice at 24 hours. MFI for CD45 in microglia was significantly increased at 24 hours in XPro1595- and etanercept-treated mice compared to saline-treated mice. Analysis of CD11b^+^CD45^high^ leukocytes showed an increase in the number of infiltrating leukocytes in all groups at 24 hours compared to non-lesioned mice. No change in MFI for CD45 in leukocytes was observed (four to six per group). **(C)** CD11b-immunostained sections six hours, 24 hours and five days after pMCAO (shown for saline-treated mice). Scale bar: 100 μm. cc: corpus callosum, IF: infarct. **(D)** Anti-TNF therapy did not affect brain *CD11b* and *iNOS* mRNA levels. Brain *IL-1β* mRNA levels were found increase at 24 hours in etanercept-treated mice, compared to six hours and five days and to saline- and XPro1595-treated mice. *Arg1* mRNA levels were found to increase in saline-treated mice at 24 hours compared to six hours and five days and in XPro1595-treated mice at 24 hours compared to five days. *IL-10* mRNA levels were found to decrease in XPro1595-treated mice 24 hours and five days compared to six hours (one-way ANOVA, four to six per group). (*P <0.05, **P <001). Arg1, arginase 1; ANOVA, analysis of variance; FSC, forward scatter; IL, interleukin; iNOS, inducible nitric oxide synthase; MFI, mean fluorescent intensity; pMCAO, permanent middle cerebral artery occlusion; SSC, side scatter; TNF, tumor necrosis factor; qPCR, quantitative polymerase chain reaction.

In order to evaluate the effect of XPro1595 and etanercept on microglial and leukocyte reactions, we investigated the number of CD11b^+^CD45^dim^ microglia and CD11b^+^CD45^high^ leukocytes in the different treatment groups after pMCAO (Figure 3A, B). The total number of CD11b^+^CD45^dim^ microglia was found to be significantly increased in all treatment groups at six and 24 hours after pMCAO, compared to non-lesioned control mice. Interestingly, when the CD11b^+^CD45^dim^ microglial population was evaluated in mice treated with either XPro1595 or etanercept and allowed to survive for 24-hours, the estimated number of CD11b^+^CD45^dim^ microglia in the lesioned cortex had increased in etanercept-treated mice, though not quite significant, and significantly in XPro1595-treated mice as compared to saline-treated mice (Figure 3B, upper left graph). Estimation of the number of infiltrating CD11b^+^CD45^high^ leukocytes showed a significant increase in all treatment groups at 24 hours compared to non-lesioned control mice, but otherwise revealed no difference among treatment groups (Figure 3B, upper right graph). The total number of CD11b^+^CD45^dim^ microglia was also found to increase at 24 hours in the contralateral cortex (*P* <0.01), but no changes were found between treatment groups (*P* >0.05, data not shown). The total number of CD11b^+^CD45^high^ leukocytes did not change in the contralateral cortex after pMCAO in any of the treatment groups (*P* >0.05, data not shown).

Since microglia and leukocytes become activated by pMCAO [19], we investigated whether the cellular level of CD45, a protein known to be involved in activation of hematopoietic cells, was affected at 24 hours. MFI analysis showed a significant increase in CD45 by microglia in XPro1595- and etanercept-treated mice as compared to saline-treated mice (Figure 3B, lower left graph); however, CD45 expression was comparable on leukocytes 24 hours after pMCAO (Figure 3B, lower right graph). Furthermore, since TNF has been directly shown to regulate CD11b expression in mouse microglial cells [26], we also investigated whether CD11b expression was affected by TNF treatment 24 hours after pMCAO. However, no differences were observed in the MFI for CD11b by microglia or leukocytes (*P* = 0.73 and *P* = 0.79, respectively, data not shown). MFI values for CD45 and CD11b did not change in the contralateral cortex for microglia (*P* = 0.94 and *P* = 0.17, respectively, data not shown) or leukocytes (*P* = 0.07 and *P* = 0.33, respectively, data not shown). These results suggest that microglial activation is increased in the ipsilateral hemisphere in anti-TNF-treated mice. Using immunohistochemistry, we found CD11b^+^ cells to be distributed similarly in all three treatment groups (Figure 3C, shown for saline only).

Based on the increased number of CD11b^+^CD45^dim^ microglia 24 hours after pMCAO in anti-TNF-treated mice, we next investigated whether anti-TNF therapy affected the phenotype of microglial activation after pMCAO. Using qPCR, we found no differences in CD11b mRNA or iNOS mRNA levels between saline- and anti-TNF-treated groups (Figure 3D). In contrast, IL-1β mRNA levels were found to be significantly increased in etanercept-treated mice compared to saline-treated mice 24 hours after pMCAO, suggesting that blocking both solTNF and tmTNF increases mRNA levels of this pro-inflammatory cytokine. Even though Arg1 mRNA levels were found to change significantly over time in saline- and XPro1595-treated mice, there were no difference between treatment groups at the different time points investigated. The same was true for IL-10 mRNA levels, which were found to change significantly over time in saline-treated mice, but no differences were observed between treatment groups. These findings suggest that etanercept treatment may induce mRNA changes associated with an M1 phenotype at 24 hours, whereas no changes were observed between treatment groups in the M2 phenotype.

### Changes in TNF levels in mice treated with anti-TNF therapy

**Figure 4:**
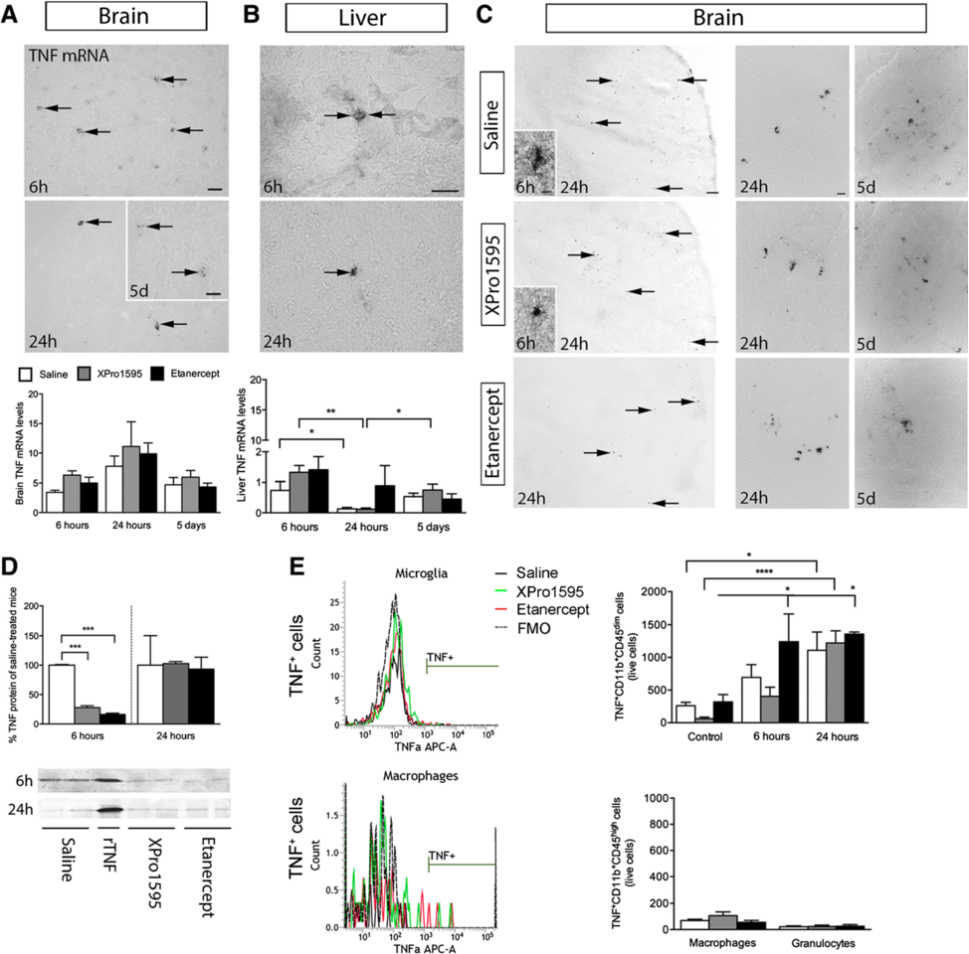
**Liver and brain TNF expression following anti-** **TNF therapy. (A)** TNF mRNA^+^ cells six hours, 24 hours and five days after pMCAO (shown for saline-treated mice). Scale bar: 30 μm. *TNF* mRNA levels increased transiently at 24 hours (four to six per group). **(B)** A few TNF mRNA^+^ cells were located in the liver preferentially at six hours (shown for saline-treated mouse), but also to some extent in etanercept-treated mice at 24 hours. Scale bar: 30 μm. Liver *TNF* mRNA levels were decreased in saline-treated mice at 24 hours compared to six hours and in XPro1595-treated mice at 24 hours compared to six hours and five days (*P <0.05, **P <0.01, three to six per group). **(C)** TNF stained sections from mice that had survived six hours (inserts), 24 hours and five days. At 24 hours and five days TNF protein expression was localized to the infarct and peri-infarct and cells displayed microglial and leukocyte morphology. Scale bars = 200 μm (low magnifications) and 20 μm (high magnifications). **(D)** Western blots for TNF levels after pMCAO demonstrating reduced TNF levels at six hours in XPro1595- (27.9% ± 3.3%) and etanercept-treated (16.2% ± 2.3%) mice compared to saline-treated mice (100% ± 1.3%) (***P <0.001, one-way ANOVA, followed by Bonferroni *post-hoc* test). At 24 hours, TNF protein levels were comparable in saline- (100% ± 50.3%), XPro1595- (102.7% ± 3.4%) and etanercept-treated (93.2% ± 20.4%) mice. **(E)** Flow cytometry profiles gated on CD11b^+^CD45^+^ cells showing primarily TNF^+^ microglia at 24 hours after pMCAO. Only few TNF^+^ macrophages and TNF^+^ granulocytes were present at 24 hours (four to six per group, **P <0.01; ****P <0.0001). ANOVA, analysis of variance; d, days; FMO, fluorescence minus one; h, hours; pMCAO, permanent middle cerebral artery occlusion; rTNF, recombinant TNF; TNF, tumor necrosis factor; qPCR, quantitative polymerase chain reaction.

TNF mRNA^+^ cells were observed within the infarct and peri-infarct in all groups after pMCAO (Figure 4A, shown for saline only). Cells were most numerous at 24 hours, with very few cells observed at five days. These findings were confirmed by qPCR, showing a transient increase in *TNF* mRNA at 24 hours (Figure 4A). As part of the acute phase response (APR) to brain injury, TNF mRNA^+^ cells were also found in the liver primarily six hours after pMCAO, as supported by qPCR analysis (Figure 4B). By 24 hours, there was a significant reduction in *TNF* mRNA transcription in the liver in saline- and XPro1595-treated mice, consistent with findings of very few TNF mRNA^+^ cells. To study whether anti-TNF therapy was capable of reducing TNF levels in the brain within the therapeutic window, we first performed immunohistochemistry on tissue from mice that had survived six hours, 24 hours and five days after pMCAO (Figure 4C). At six hours, TNF^+^ microglial and leukocyte-like cells were located within the infarct and in the peri-infarct in saline-treated mice six hours after pMCAO (inserts in Figure 4C), and rarely observed in anti-TNF-treated mice. At 24 hours and five days, cells were found to be located in the infarct and in the peri-infarct (low magnifications in Figure 4C, shown for 24 hours only) in all treatment groups. The cells were found to have microglial- and leukocyte-like morphology (high magnifications in Figure 4C). In order to support the findings of reduced TNF^+^ cells at six hours in anti-TNF-treated mice, we performed Western blotting for TNF on brain tissue from mice allowed to survive for six- and 24-hours (Figure 4D). We found reduced TNF levels at six hours in XPro1595-treated mice, and even more so in etanercept-treated mice, as compared to saline-treated mice, suggesting that both types of anti-TNF therapies were capable of reducing TNF availability within six hours after pMCAO. At 24 hours, comparable TNF levels were found by Western blotting, supporting the immunohistochemistry data.

Using flow cytometry, we further investigated changes in the number of TNF producing microglia (TNF^+^CD11b^+^CD45^dim^ cells), macrophages (TNF^+^CD11b^+^CD45^high^Gr1^−^ cells) and granulocytes (TNF^+^CD11b^+^CD45^high^Gr1^+^ cells) six and 24 hours after pMCAO compared to non-lesioned control mice (Figure 4E). We found a tendency towards a reduced number of TNF^+^CD11b^+^CD45^dim^ microglia in XPro1595-treated non-lesioned control mice and mice allowed to survive for six hours, however this was not significant (Figure 4E, upper panel). At 24 hours, the number of TNF^+^CD11b^+^CD45^dim^ microglia had increased significantly in all treatment groups compared to non-lesioned control mice, with no differences between treatment groups (Figure 4E). TNF^+^ macrophages (TNF^+^CD11b^+^CD45^high^Gr1^−^ cells) and TNF^+^ granulocytes (TNF^+^CD11b^+^CD45^high^Gr1^+^ cells) (Figure 4E, lower panel) were not detected by flow cytometry in the ischemic infarct until 24 hours after pMCAO and at this time point there were no differences between treatment groups. Note that at 24 hours, the total number of TNF-producing leukocytes was significantly lower than the number of TNF-producing microglia (Figure 4E, please compare upper graph with lower graph).

### Anti-TNF therapy affects granulocyte infiltration into the infarct 24 hours after focal cerebral ischemia

**Figure 5:**
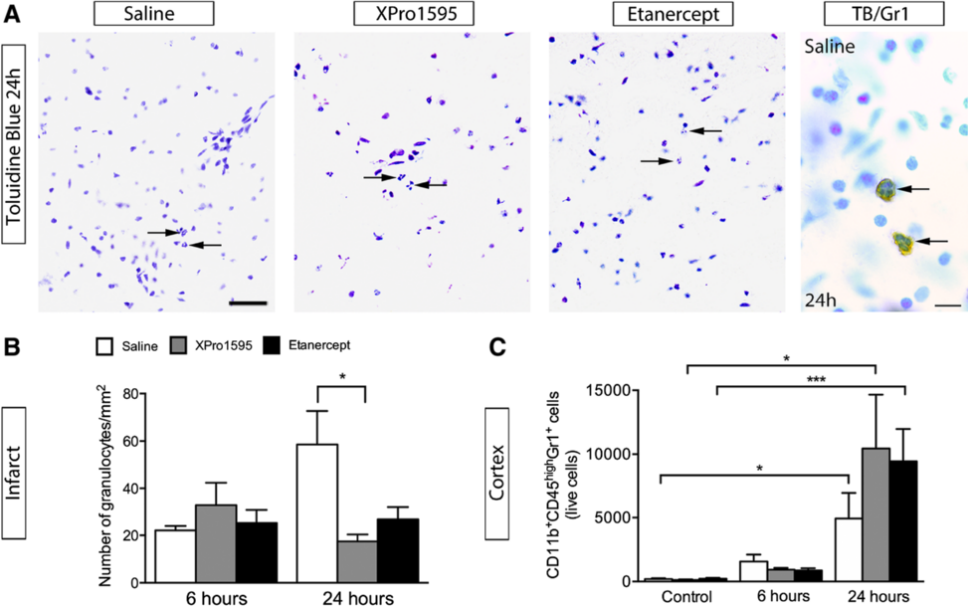
**The effect of anti-** **TNF therapy on the number of granulocytes in the infarct. (A)** Representative photomicrographs of TB-stained brain sections from saline-, XPro1595-, and etanercept-treated mice allowed to survive for 24 hours after focal cerebral ischemia, demonstrating infiltration of polymorphonucleated cells into the ischemic infarct. Co-localization of polymorphonucleated cells in TB-stained sections with a granulocyte (Gr1) marker was verified using immunohistochemistry in saline-treated mice allowed to survive for 24 hours. Scale bars: left 30 μm and right 10 μm. **(B)** Estimation of the number of infiltrating polymorphonucleated cells per mm^2^ within the infarct showed a significantly increased number of cells in saline-treated mice (58.6 ± 14.1 granulocytes/mm^2^) compared to XPro1595-treated mice (17.4 ± 3.0 granulocytes/mm^2^) 24 hours after ischemia (*P <0.05, one-way ANOVA, followed by Bonferroni *post-hoc* test; four to eight per group). **(C)** Flow cytometry analysis of the number of infiltrating CD11b^+^CD45^high^Gr1^+^ cells in the ipsilateral neocortex of non-lesioned control mice and mice six and 24 hours after pMCAO showed a significant increase in the total number of infiltrating granulocytes in saline-, XPro1595- and etanercept-treated mice at 24 hours compared to non-lesioned control mice. No difference between treatment groups was observed (four to six per group). ANOVA, analysis of variance; h, hours; pMCAO, permanent middle cerebral artery occlusion; TB, toluidine blue; TNF, tumor necrosis factor.

As TNF facilitates granulocyte infiltration into the CNS [27], we counted the number of granulocytes in the infarct six and 24 hours after pMCAO (Figure 5). Based on their characteristic nuclear morphology (between two and five lobes), which was further verified using an anti-Gr1 antibody (Figure 5A, left), we observed comparable numbers/mm^2^ of granulocytes six hours after pMCAO, but increased numbers/mm^2^ in saline-treated mice 24 hours after pMCAO compared to anti-TNF-treated mice (Figure 5B). Using flow cytometry, we found a significant increase in the number of CD11b^+^CD45^high^Gr1^+^ granulocytes at 24 hours compared to non-lesioned control mice, however the number was comparable in the whole ipsilateral cortex in all three groups at all time points investigated (Figure 5C).

### Anti-TNF therapy affects the liver acute phase response after focal cerebral ischemia

**Figure 6:**
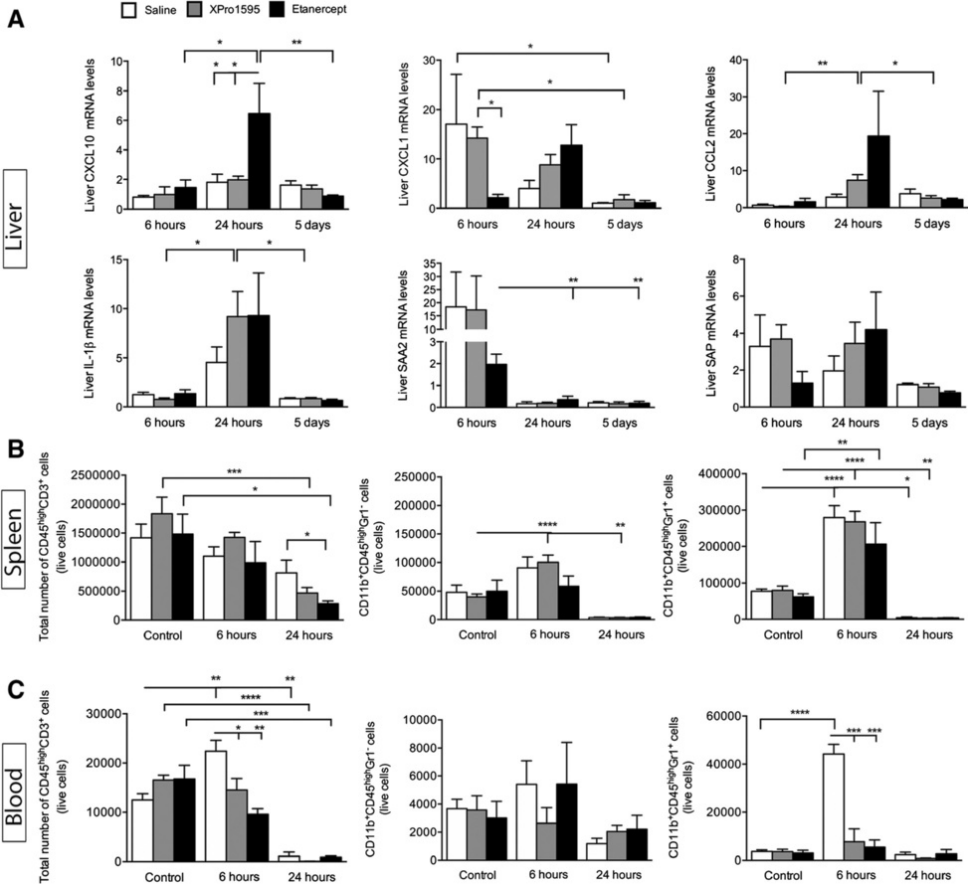
**The effect of anti-** **TNF on the APR after focal cerebral ischemia. (A)** Changes in liver *CXCL10*, *CXCL1*, *CCL2*, *IL-1β*, *SAA2* and *SAP* mRNA levels after pMCAO (*P <0.05, **P <0.01, one-way ANOVA followed by Bonferroni *post-hoc* test, three to six per group). **(B)** Flow cytometry analysis of spleen samples six and 24 hours after pMCAO show a decrease in numbers of T-cells (CD45^+^CD3^+^) in XPro1595- and etanercept-treated mice 24 hours after pMCAO compared to non-lesioned mice. At 24 hours, the number of T-cells were decreased in etanercept-treated mice compared to saline-treated mice. The number of monocytes (CD11b^+^CD45^high^Gr1^−^) was found to increase in XPro1595-treated mice at six hours and decrease at 24 hours, compared to non-lesioned mice. The number of granulocytes (CD11b^+^CD45^high^Gr1^+^) was found to increase at six hours in all groups compared to non-lesioned mice, and to decrease at 24 hours in saline- and XPro1595-treated mice. **(C)** Flow cytometry analysis of blood samples showed that the number of T-cells increased at six hours in saline-treated, but not in XPro1595- and etanercept-treated mice, compared to non-lesioned mice. The total number of T-cells was increased in saline-treated mice compared to XPro1595- and etanercept-treated mice at six hours. At 24 hours, the total number of T-cells was decreased in all treatment groups. No change was observed at any time point in blood monocytes. The number of granulocytes was found to increase in blood in saline-treated mice at six hours compared to non-lesioned mice, but also compared to XPro1595- and etanercept-treated mice with six hours survival (*P <0.05, four to six per group). ANOVA, analysis of variance; APR, acute phase response; CCL, chemokine (C-C motif) ligand; CXCL, chemokine (C-X-C motif) ligand; IL, interleukin; pMCAO, permanent middle cerebral artery occlusion; SAA2, serum amyloid A2; SAP, serum amyloid P; TNF, tumor necrosis factor.

Since anti-TNF therapy improved functional outcome without affecting infarct size, and since suppression of the APR has previously been shown to correlate with improvements in behavior in motivational tests [28], we analyzed the peripheral APR. Hepatic expression of chemokine ligand *CXCL10*, involved in monocyte and macrophage and natural killer (NK) cell infiltration, was significantly altered by etanercept at 24 hours compared to six hours and five days after pMCAO (Figure 6A). *CXCL10* mRNA levels in saline- and XPro1595-treated mice remained relatively constant over time, whereas we observed a significant increase in etanercept-treated mice. Since we observed no effect of blocking only solTNF on *CXCL10* mRNA, these data suggest a close interplay between tmTNF and *CXCL10* mRNA regulation in the liver.

At six hours after pMCAO, mRNA levels of hepatic *CXCL1*, primarily involved in granulocyte infiltration, were significantly lower in etanercept-treated mice compared to XPro1595-treated mice (Figure 6A). At five days, we observed an overall drop in *CXCL1* mRNA in saline- and XPro1595-treated mice, but not in etanercept-treated mice. These data suggest that liver *CXCL1* mRNA expression is affected differently by tmTNF and solTNF after pMCAO. Hepatic *CCL2*, a chemokine primarily referred to as monocyte chemotactic protein, mRNA levels were only affected in XPro1595-treated mice, which displayed a transient increase 24 hours after pMCAO compared to both six hours and five days (Figure 6A), suggesting that solTNF plays a role in recruitment of monocytes into the liver. The mRNA levels of *IL-1β*, which is known to be involved in neurotoxicity after focal cerebral ischemia [1], were significantly increased 24 hours after pMCAO in anti-TNF-treated mice (Figure 6A), but there were no differences between saline-, XPro1595- and etanercept-treated mice.

Two late-phase APR proteins, serum amyloid A2 (SAA2) and serum amyloid P-component (SAP), both of which are known to be regulated by proinflammatory cytokines such as TNF and IL-1β, were also investigated (Figure 6A). Anti-TNF therapy following pMCAO did not influence liver *SAA2* or *SAP* mRNA levels. Liver *SAP* mRNA levels did not differ at any time point after pMCAO. Correlation analyses of liver chemokine expression after pMCAO are presented in Additional file 1: Table S1.

Flow cytometric analyses of spleen leukocyte populations (Figure 6B) showed that the number of CD45^+^CD3^+^ T-cells in the spleen was significantly decreased in XPro1595- and etanercept-treated mice 24 hours after pMCAO compared to non-lesioned control mice. Furthermore, the number of T-cells was significantly decreased in etanercept-treated mice compared to saline-treated mice at 24 hours (Figure 6B). These findings can possibly be explained by reports of increased caspase-induced apoptosis induction of T-cells in the spleen following treatment with anti-TNF therapies, such as infliximab [29]. In the spleen we also found significant changes in the total number of spleen CD11b^+^CD45^high^Gr1^−^ monocytes in XPro1595-treated mice, with a significant increase at six hours and a significant decrease at 24 hours compared to non-lesioned XPro1595-treated control mice (Figure 6B). No changes were observed in saline- or etanercept-treated mice and no significant difference was observed between groups. Also, the total number of spleen CD11b^+^CD45^high^Gr1^+^ granulocytes changed significantly over time compared to non-lesioned control mice. There was as significant increase in all groups of mice at six hours and a significant decrease in saline- and XPro1595-treated mice at 24 hours (Figure 6B). Flow cytometric analyses of blood leukocyte populations (Figure 6C) showed a significant increase in circulating T-cells in saline-treated mice at six hours and a significant decrease in all treatment groups at 24 hours compared to non-lesioned control mice. At six hours, the total number of circulating T-cells was also significantly increased in saline-treated mice compared to both XPro1595- and etanercept-treated mice. No changes were observed in the total number of circulating blood monocytes at any time point investigated, or between treatment groups. In the blood, the total number of circulating granulocytes significantly increased in saline-treated mice compared to both non-lesioned control mice and compared to XPro1595- and etanercept-treated mice allowed to survive for six hours. These results demonstrated that anti-TNF therapy decreased the total number of circulating T-cells and granulocytes early (six hours) after pMCAO.

### Anti-TNF therapy impacts microvesicle size and number after focal cerebral ischemia

**Figure 7:**
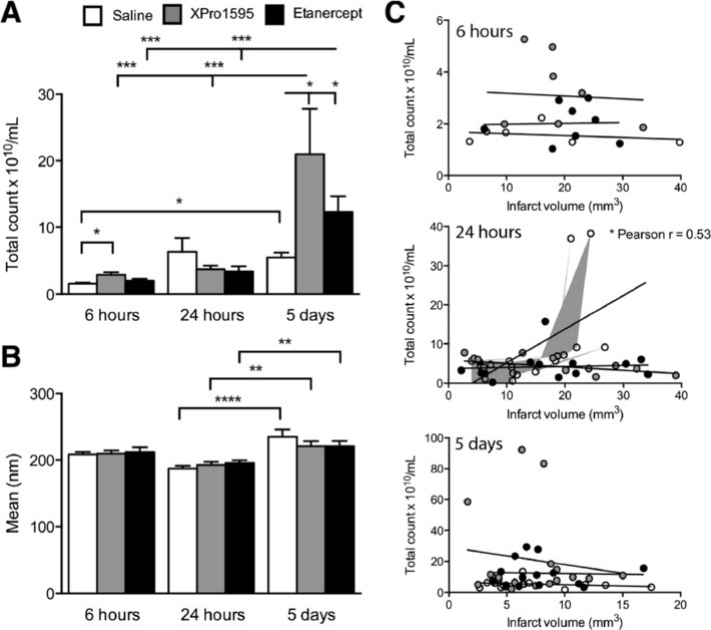
**The effect of anti-** **TNF therapy on microvesicle counts and size. (A)** Estimations of the total numbers of microvesicles after focal cerebral ischemia showing altered counts with anti-TNF therapy. **(B)** Estimation of the mean diameter of microvesicles after focal cerebral ischemia. **(C)** Correlation analysis of the total count of microvesicles and the infarct size at six hours (top), 24 hours (middle) and five days (bottom) after focal cerebral ischemia showing a significant correlation in saline-treated mice at 24 hours. Area fill for 24 hours is shown in grey (*P <0.05; **P <0.01; ***P <0.001, ****P <0.0001, Pearson r correlation analysis and one-way ANOVA, followed by Bonferroni *post-hoc* test; three to 24 per group). mm, millimeter; nm, nanometer; TNF, tumor necrosis factor.

As microvesicle number and infarct size have been shown to correlate and possibly be an indicator of inflammation, we analyzed microvesicle number and size after pMCAO. Comparisons showed similar numbers in saline- (2.7 ± 0.5 × 10^10^/ml) and anti-TNF-treated control mice (XPro1595: 3.3 ± 0.2 × 10^10^/ml and etanercept 3.2 ± 0.8 × 10^10^/ml). By six hours, we found significantly more microvesicles in XPro1595-treated mice compared to saline-treated mice (Figure 7A), an effect which was observed in both XPro1595- and etanercept-treated mice five days after pMCAO. When investigating temporal changes, we observed significant increases in microvesicle numbers in all groups five days after pMCAO compared to survival for six hours.

Overall, the mean diameter of microvesicles changed over time, but not within treatment groups. There was a general increase in the mean size at six hours compared to controls (saline: 170.8 ± 5.3 nm; XPro1595: 170.0 ± 6.5 nm; and etanercept: 190.0 ± 5.0 nm; *P* <0.05) and five days after pMCAO, compared to 24 hours after pMCAO (Figure 7B). The difference in microvesicle size over time most likely reflects differences in origin. Interestingly, at 24 hours we observed a small yet significant correlation between microvesicle numbers and infarct volume in saline-treated mice, but not in anti-TNF-treated mice (Figure 7C). This correlation was not observed at any other time point.

## Discussion

In the present study, we found that in a mouse model of focal cerebral ischemia, two different TNF inhibitors improved functional outcome, modified the hepatic APR, changed microglial CD45 expression in the neocortex and affected microvesicle numbers in the serum, without affecting lesion volume. Selective inhibition of solTNF using XPro1595 had comparable effects with non-selective inhibition of both solTNF and tmTNF using etanercept, suggesting that solTNF plays an important role in the observed effects.

The finding that anti-TNF therapy did not have an effect on lesion volume, but improved behavioral outcome in the present study, has previously been shown in an animal model of hemorrhagic stroke where they used etanercept [30]. Also, in a model of transient focal cerebral ischemia in mice, Sumbria *et al*. [31] showed that systemic injection of etanercept had no effect on lesion volume. We also recently showed that anti-TNF therapy had no effect on lesion size after spinal cord injury when administered systemically, however when anti-TNF therapies were administered epidurally for three consecutive days using mini-osmotic pumps, we observed both a reduction in lesion size and an improvement in functional outcome in XPro1595-treated mice, but not in etanercept-treated mice [12]. These findings suggest that anti-TNF therapies have to be administered directly to the lesioned CNS in order to affect lesion size. Recent findings that systemically administered XPro1595 (10 mg/kg) does indeed cross the blood-brain barrier (BBB) [32] and the findings in the present study that CNS TNF levels are reduced six hours after pMCAO in anti-TNF-treated mice suggest that both etanercept and XPro1595 may have crossed the BBB, however this could also be the result of endothelial dysfunction rather than transport across the BBB [33]. Future studies using mini-osmotic pumps following experimental stroke are needed in order to clarify whether XPro1595 can also reduce lesion size after experimental stroke.

One-way etanercept has been shown to improve behavior is through anti-nociceptive effects. Boettger *et al*. showed improved locomotor and pain-related behavior after etanercept treatment in a rat model of chronic antigen-induced arthritis, even with no resolution in joint swelling and inflammation, and suggested that reduction of the effect of peripheral TNF on pain fibers contributed to pain relief [34]. This could also be the case in the present study, however, this will require further investigation. The suppression of granulocyte recruitment to experimental stroke lesions has previously been shown to reduce infarct volume and reduce cell death, but this has usually been linked to associated reductions in infarct volume [35]. A reduction of granulocyte infiltration into the lesioned brain in animals treated with etanercept has been suggested to be mediated via etanercept’s effect on the APR in the liver [28]. In the present study, we found that anti-TNF therapy altered the APR in the liver, and more specifically the mRNA expression of chemokines associated with granulocyte recruitment, such as CXCL10 and CXCL1.

Systemic injections of etanercept have also previously been found to attenuate traumatic brain injury by ameliorating neurological and motor dysfunction and by initially reducing brain TNF protein levels [36,37]. Despite unaltered brain TNF mRNA levels and comparable numbers of TNF^+^ microglia and leukocytes at 24 hours in the different experimental groups, we found that brain TNF protein levels were decreased six hours after pMCAO in mice treated with anti-TNF therapy, which is in line with the mechanism of action of both XPro1595 and etanercept neutralizing TNF at the protein level. In the present study, TNF^+^ cells were located in the infarct and peri-infarct at six hours in all three experimental groups, but to a much lesser extent in XPro1595- and etanercept-treated mice than in saline-treated mice. Furthermore, the morphology of TNF^+^ cells in XPro1595- and etanercept-treated mice were more glial-like, whereas the morphology in saline-treated mice were mixed glial-like and macrophage-like, suggesting that the reduced levels of TNF in the anti-TNF-treated mice could be due to a peripheral reduction in TNF produced by infiltrating macrophages.

Etanercept has been suggested to ameliorate microglial activation [38], however, in the present study, we found no effect of anti-TNF therapy on CD11b expression, whereas microglial, but not macrophage, CD45 expression was increased in the ipsilateral hemisphere 24 hours after anti-TNF treatment. This, combined with the increased number of CD11b^+^CD45^dim^ microglia 24 hours after pMCAO, suggests that anti-TNF therapy either increases microglial proliferation in the brain or increases the surface expression of the CD45 marker on microglia as a response to treatment in the ischemic brain. Previous studies have shown that increased CD45 expression is involved in ‘microglial alertness’ and activation following injury to the CNS, and that an increase in this surface protein likely reflects a response to ongoing neuroinflammation [39], suggesting that anti-TNF treatment may induce increased activation of microglia.

Microvesicles have been described as important mediators of intercellular communication and are emerging as potential biomarkers of tissue damage. Interestingly, microvesicles have been found to propagate inflammatory signals [40], and the subtypes of endothelial microvesicles from stroke patients have been shown to correlate with lesion volume and functional outcome [41]. Importantly, in the present study, microvesicle numbers in saline-treated mice 24 hours after pMCAO were found to correlate significantly with infarct volume, which was not the case in either etanercept- or XPro1595-treated mice, suggesting an altered response due to anti-TNF therapy. In line with this, we observed increased numbers of microvesicles in anti-TNF-treated mice five days after pMCAO. The microvesicles showed characteristics of shed microvesicles due their mean diameter of 200 nm [42]. As the increase in microvesicle number does not appear to correlate to the infarct volume in anti-TNF-treated groups, we speculate that the vesicles contribute to an altered inflammatory response in the brain, as microvesicles represent an important means of intercellular communication between cells, serving as transfer vehicles for proteins, lipids, RNA and microRNA [42]. In addition, microvesicles are known to exchange information with endothelium, thereby actively regulating vascular function or participating in vascular rearrangement [43]. However, their precise functions are still not fully understood.

Based on the data presented here, we suggest that XPro1595 and etanercept improve functional outcome in mice subjected to focal cerebral ischemia by altering the peripheral immune response leading to decreased infiltration of granulocytes into the infarct, potential altered microglial alertness and, most likely, an improvement in motivational state. The finding that XPro1595 was just as efficient as etanercept in improving functional outcome and altering the APR suggests that solTNF (and not tmTNF) is principally involved in peripheral inflammation after stroke. Finally, previous studies have indicated a direct correlation between TNF and blood pressure in hypertensive humans [44] and have reported a decrease in blood pressure in an experimental model of systemic lupus erythematosis, a chronic inflammatory disorder with prevalent hypertension, following etanercept administration weekly for a duration of four weeks [45]. In the present study, we only administered etanercept once, 30 minutes after induction of experimental stroke, which did not appear to affect either the mortality or health status of the mice, however, whether etanercept did indeed reduce blood pressure in the present study remains to be elucidated in future studies.

All together, these findings may have important implications for future treatments of solTNF-mediated diseases, where anti-TNF therapy targeting both solTNF and tmTNF can be substituted with drugs only targeting solTNF, potentially resulting in less severe side effects for the patient, including demyelinating diseases and infections.

## Additional file

Additional file 1: Table S1. Pearson r correlation analysis of liver chemokine mRNA in saline-, XPro1595- and etanercept-treated mice six hours, 24 hours and five days after focal cerebral ischemia.

## Abbreviations

APR: Acute phase response
BBB: Blood–brain-barrier
CCL2: Chemokine (C-C motif) ligand 2
CXCL: Chemokine (C-X-C motif) ligand
DN-TNF: Dominant-negative tumor necrosis factor
GAPDH: Glyceraldehyde phosphate dehydrogenase
IL-1β: Interleukin-1 beta
MCA: Middle cerebral artery
MFI: Mean fluorescence intensity
PBS: Phosphate-buffered saline
PFA: Paraformaldehyde
pMCAO: permanent middle cerebral artery occlusion
RM: Repeated measures
Rpm: rotations per minute
SAA2: Serum amyloid A2
SAP: Serum amyloid P-component
solTNF: soluble tumor necrosis factor
TACE: Tumor necrosis factor-alpha converting enzyme
TB: Toluidine blue
TBI: Traumatic brain injury
tmTNF: transmembrane tumor necrosis factor
TNF: Tumor necrosis factor
