# Supplementary material for: Systemically administered anti-TNF therapy ameliorates functional outcomes after focal cerebral ischemia
Source: J Neuroinflammation. 2014 Dec 12;11:203. doi: 10.1186/s12974-014-0203-6 (PMC4272527; doi:10.1186/s12974-014-0203-6)
Supplement: Additional file 1: Table S1. — Pearson r correlation analysis of liver chemokine mRNA in saline-, XPro1595- and etanercept-treated mice six hours, 24 hours and five days after focal cerebral ischemia. [file 12974_2014_203_MOESM1_ESM.docx]

| Pearson (r) | **Saline** | **XPro1595** | **Etanercept** |
| --- | --- | --- | --- |
| **6 hours** | | | |
| CXCL1 mRNA vs. CXCL10 mRNA | P = 0.54  r = 0.46 (R^2^ = 0.21) | P = 0.48  r = -0.73 (R^2^ = 0.53) | P = 0.29  r = -0.52 (R^2^ = 0.27) |
| CXCL1 mRNA vs. CCL2 mRNA | P = 0.45  r = 0.55 (R^2^ = 0.30) | P = 0.10  r = 0.99 (R^2^ = 0.98) | P = 0.22  r = -0.58 (R^2^ = 0.34) |
| CXCL10 mRNA vs. CCL2 mRNA | **P = 0.05**  **r = 0.94 (R^2^ = 0.90)** | P = 0.59  r = -0.61 (R^2^ = 0.37) | ******P < 0.0001**  **r = 1.00 (R^2^ = 0.99)** |
| **24 hours** | | | |
| CXCL1 mRNA vs. CXCL10 mRNA | ****P < 0.01**  **r = 0.94 (R^2^ = 0.88)** | P = 0.19  r = 0.62 (R^2^ = 0.39) | P = 0.75  r = 0.75 (R^2^ = 0.56) |
| CXCL1 mRNA vs. CCL2 mRNA | P = 0.07  r = 0.77 (R^2^ = 0.60) | **P = 0.05**  **r = 0.81 (R^2^ = 0.65)** | P = 0.15  r = 0.75 (R^2^ = 0.56) |
| CXCL10 mRNA vs. CCL2 mRNA | P = 0.12  r = 0.71 (R^2^ = 0.50) | ***P < 0.05**  **r = 0.90 (R^2^ = 0.81)** | ****P < 0.01**  **r = 0.97 (R^2^ = 0.95)** |
| **5 days** | | | |
| CXCL1 mRNA vs. CXCL10 mRNA | ****P < 0.01**  **r = 0.97 (R^2^ = 0.94)** | P = 0.46  r = -0.38 (R^2^ = 0.14) | P = 0.60  r = -0.27 (R^2^ = 0.08) |
| CXCL1 mRNA vs. CCL2 mRNA | P = 0.22  r = 0.58 (R^2^ = 0.34) | P = 0.40  r = -0.42 (R^2^ = 0.18) | P = 0.97  r = -0.02 (R^2^ = 0.00) |
| CXCL10 mRNA vs. CCL2 mRNA | P = 0.29  r = 0.52 (R^2^ = 0.27) | P = 0.40  r = 0.43 (R^2^ = 0.18) | P = 0.88  r = -0.08 (R^2^ = 0.01) |

Supplemental Table 1. Pearson r correlation analysis of liver chemokines in saline-, XPro1595- and etanercept-treated mice 6 hours, 24 hours and 5 days after focal cerebral ischemia.
